# Surface Functionalization of an Aluminum Alloy to Generate an Antibiofilm Coating Based on Poly(Methyl Methacrylate) and Silver Nanoparticles

**DOI:** 10.3390/molecules23112747

**Published:** 2018-10-24

**Authors:** Lisa Muñoz, Laura Tamayo, Miguel Gulppi, Franco Rabagliati, Marcos Flores, Marcela Urzúa, Manuel Azócar, Jose H. Zagal, María V. Encinas, Xiaorong Zhou, George Thompson, Maritza Páez

**Affiliations:** 1 Departamento de Química de los Materiales, Facultad de Química y Biología, Universidad de Santiago de Chile, Av. L. B. O‘Higgins 3363, Casilla 40, Correo 33, Santiago 9170022, Chile; miguel.gulppi.c@usach.cl (M.G.); franco.rabagliati@usach.cl (F.R.); manuel.azocar@usach.cl (M.A.); jose.zagal@usach.cl (J.H.Z.); maria.encinas@usach.cl (M.V.E.); 2 Departamento de Química, Facultad de Ciencias, Universidad de Chile, Las Palmeras 3425, Ñuñoa, Santiago 7800024, Chile; lauratamayo26@gmail.com (L.T.); maurzua@uchile.cl (M.U.); 3 Laboratory of Surface and Nanomaterials, Physics Department, Faculty of Physics and Mathematics Science, Universidad de Chile, Beauchef 850, Santiago 8370415, Chile; mflorescarra@ing.uchile.cl; 4 Corrosion and Protection Centre, School of Materials, The University of Manchester, Manchester M13 9PL, UK; xiaorong.zhou@manchester.ac.uk (X.Z.); george.thompson@manchester.ac.uk (G.T.)

**Keywords:** coating, antibiofilm, functionalization, poly(methyl methacrylate), aluminum alloy

## Abstract

An experimental protocol was studied to improve the adhesion of a polymeric poly(methyl methacrylate) coating that was modified with silver nanoparticles to an aluminum alloy, AA2024. The nanoparticles were incorporated into the polymeric matrix to add the property of inhibiting biofilm formation to the anticorrosive characteristics of the film, thus also making the coating antibiocorrosive. The protocol consists of functionalizing the surface through a pseudotransesterification treatment using a methyl methacrylate monomer that bonds covalently to the surface and leaves a terminal double bond that promotes and directs the polymerization reaction that takes place in the process that follows immediately after. This results in more compact and thicker poly(methyl methacrylate) (PMMA) coatings than those obtained without pseudotransesterification. The poly(methyl methacrylate) matrix modified with nanoparticles was obtained by incorporating both the nanoparticles and the methyl methacrylate in the reactor. The in situ polymerization involved combining the pretreated AA2024 specimens combined with the methyl methacrylate monomer and AgNps. The antibiofilm capacity of the coating was evaluated against *P. aeruginosa*, with an excellent response. Not only did the presence of bacteria decrease, but the formation of the exopolymer subunits was 99.99% lower than on the uncoated aluminum alloy or the alloy coated with unmodified poly(methyl methacrylate). As well and significantly, the potentiodynamic polarization measurements indicate that the PMMA-Ag coating has a good anticorrosive property in a 0.1-M NaCl medium.

## 1. Introduction

A coating that protects a surface has two interfacial regions; one faces the metal surface, namely the metal-coating interface, and the other faces the environment, namely the coating-environment interface. In the first, surface-coating adhesion plays a determining role, while in the second, the adhesion of environmental species (chemical and biological) to the surface of the coating is crucial. Influencing the two interfaces can enhance the durability of the coating and extend the useful life of the coated surface. There are several factors that affect adhesion and involve, on the one hand, the composition and morphology of the metal surface, which is closely related to the metallurgical history of the metal substrate [1,2], and on the other hand, the surface pretreatments of the metal before applying the coating [3,4].

Various treatments have been developed to improve adhesion on aluminum surfaces. The most widely used is conversion coating, which is based on chromate species. However, this approach is subject to increasingly stringent environmental regulations, which has spurred efforts to develop alternatives; these efforts have not had good results to date [5,6]. Coating adhesion is not only an issue with aluminum alloys; attempts have been made to improve the adhesion of coatings on other metal surfaces such as zinc. In fact, a recent publication reports using vinyltrimethoxysilane (VTS) as a pretreatment to functionalize oxidized zinc substrates. The functionalized zinc was then subjected to the thermally initiated copolymerization of methyl methacrylate (MMA) in the presence or absence of crosslinkers, resulting in a coating of poly(methyl methacrylate) [PMMA]. Although PMMA coatings have high levels of mechanical strength, thermal stability, and corrosion resistance, cathodic delamination is evident over time due to the hydrolysis of the siloxane bond in the inorganic polymer matrix [7,8]. There is currently major interest in research into directly bonding coatings to surfaces [7,8,9,10].

In relation to the coating environment, a series of factors have been studied to inhibit or limit the interaction of coatings with environmental species [11]; among these is the effect of the hydrophilic or hydrophobic character on the anti-adhesive performance of polymer coatings [12,13].

Considering the harmful effects of bacterial colonization and the potential applications of coatings in other areas, such as biomedicine, we wanted to expand the properties and applications of PMMA coating. PMMA is a polymer that is widely used in implantable medical devices due to its biocompatible characteristics, low inflammatory reaction, and easy processability. PMMA also has good resistance to ultraviolet light and to attack by chemical agents that are widely used in the manufacture of pneumatic actuators for pumping fluids [14]. However, PMMA is highly susceptible to bacterial colonization [15]. Faced with this problem, antibiotics have been incorporated into implants, which has been a widely-used method to prevent bacterial colonization and infection. However, the ability of bacteria to resist antibiotics poses major problems with respect to their use [16]. In this respect, new antibacterial substances that minimize bacterial resistance represent an alternative strategy. Metallic nanoparticles of silver, copper, and gold are being used as an alternative to antibiotics because of their excellent antibacterial capacity [17,18,19], broad spectrum of effectiveness, and low bacterial resistance [20]. The incorporation of nanoparticles in different polymer matrices has allowed the development of new antibacterial materials with excellent results in implants, medical devices, and membranes for water treatment [21,22].

This work proposes an experimental protocol for covalently bonding a PMMA-AgNps coating to an aluminum alloy surface. The protocol is termed pseudotransesterification, since it involves the esterification of the prehydroxylated metal surface, to finally bond the monomer methyl methacrylate (MMA) and then grow an AgNp-modified film by radical polymerization to prevent the delamination of the polymeric film and adhesion of microorganisms.

## 2. Results and Discussion

### 2.1. Characterization of the Functionalized Aluminum Alloy Surface

**Figure 1:**
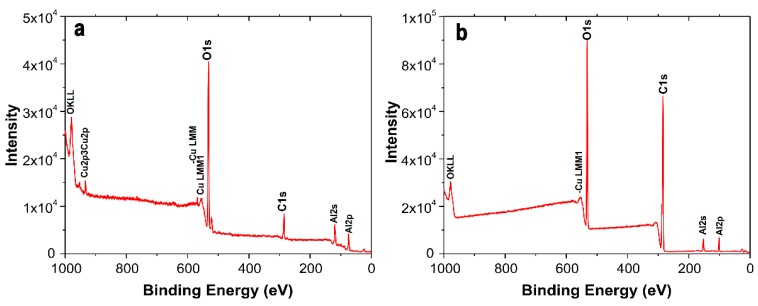
X-ray photoelectron spectroscopy (XPS) spectra of the differently pretreated AA2024 aluminum alloy: (**a**) chemically etched (AA); (**b**) chemically etched and pseudotransesterification treated (AA-TE).

**Figure 2:**
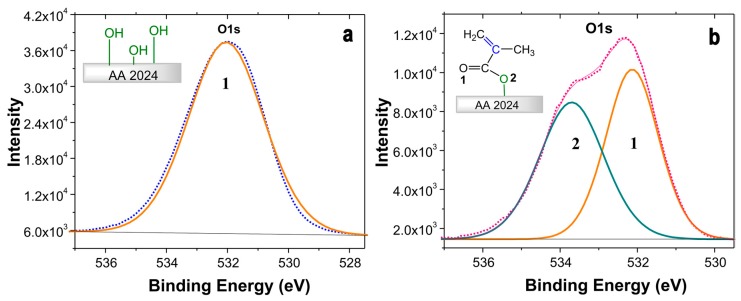
XPS spectra showing the O1s peaks on the aluminum alloy: (**a**) chemically etched (AA); (**b**) chemically etched and pseudotransesterification treated (AA-TE).

X-ray photoelectron spectroscopy (XPS) was used to characterize the AA2024 aluminum alloy after the pseudotransesterification treatment. The XPS spectra are shown in Figure 1 and Figure 2.

Figure 1 shows the survey XPS spectra of chemically etched (termed hereafter AA) and pseudotransesterification treated (termed hereafter as AA-TE) AA2024 aluminum alloy. The effect of pseudotransesterification is reflected in the significantly decreased value of the O1s:C1s intensity peaks. The ratio with pseudotransesterification is 2:3, while it is 1:5 without the treatment. This result was expected, since the presence of the methyl methacrylate monomers on the surface increases the photoelectron yields of carbon species. Changes were also expected in the oxygen peak O1s, where the hydroxyl group (-OH), which is located at 532.1 (eV) [23], participates in the modification reaction by exchanging the methoxy (-OCH_3_) group of the methyl methacrylate monomer with the Al-OH group on the metal surface. The deconvolution of the high-resolution O1s spectra of samples AA and AA-TE is shown in Figure 2.

Figure 2a shows the fit of the O1s peak on the surface of the chemically etched aluminum alloy, revealing the OH group at 531.9 eV, corresponding to the hydroxylated alumina. In contrast, Figure 2b shows the deconvolution of the O1s peak obtained from the aluminum alloy with pseudotransesterification treatment, revealing two O1s peaks. Peak 1 represents the oxygen of the carboxyl group (532.1 eV) that is present in methyl methacrylate [24], where the unreacted hydroxyl groups (-OH) appear to contribute to the intensity. Peak 2, at 533.6 eV, represents the oxygen that is bonded directly to aluminum.

In order to verify the pseudotransesterification of the aluminum alloy surface, we used α-alumina powder as the benchmark for the surface oxide layer on the aluminum alloy. Through the E/I curves obtained by differential pulse voltammetry, we observed that the alumina colloid with the pseudotransesterification treatment presented two current peaks at −0.51 and −0.83 V, which are associated with the reduction of carbonyl carbon, demonstrating that pseudotransesterification occurred on α-alumina particulates. These peaks were not observed for the colloidal system containing alumina without the pseudotransesterification treatment (see Supplementary Materials).

### 2.2. Morphological Characterization of the Coated Aluminum Alloy

**Scheme 1:**
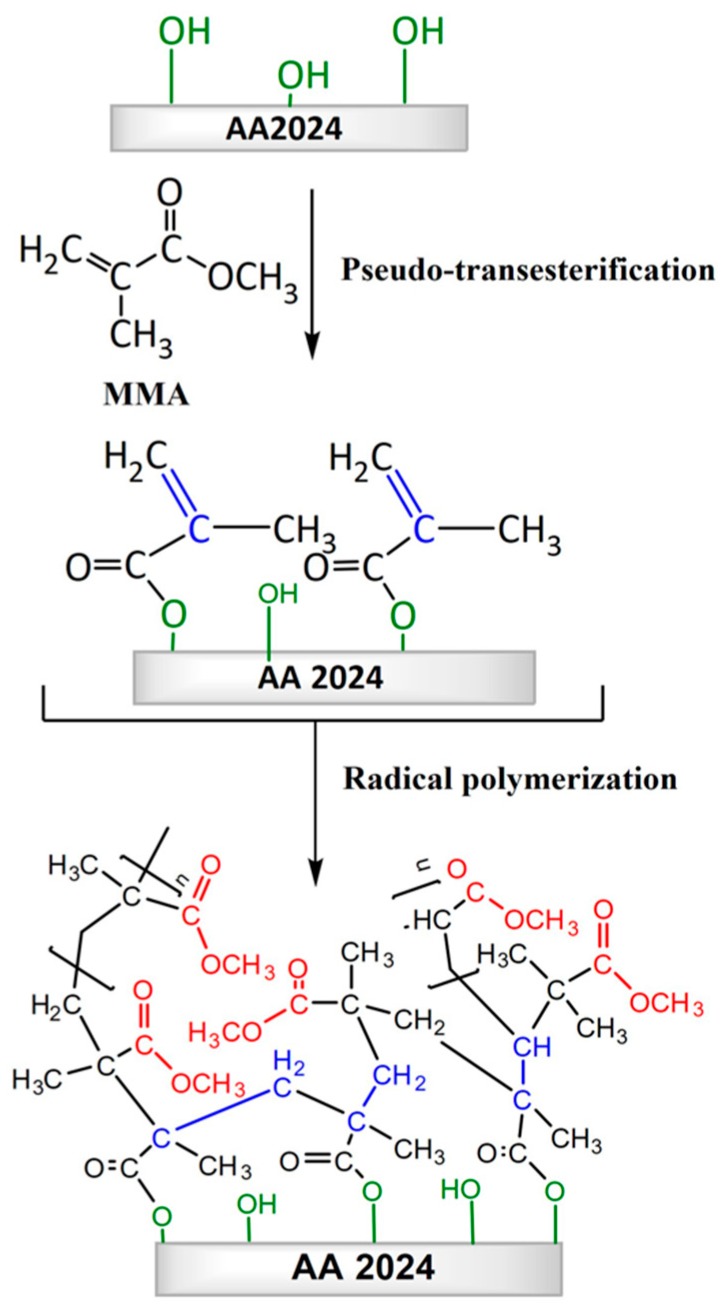
Scheme of the surface modification steps.

Following the pseudotransesterification treatment, the PMMA coating was achieved by mass radical polymerization (Scheme 1).

**Figure 3:**
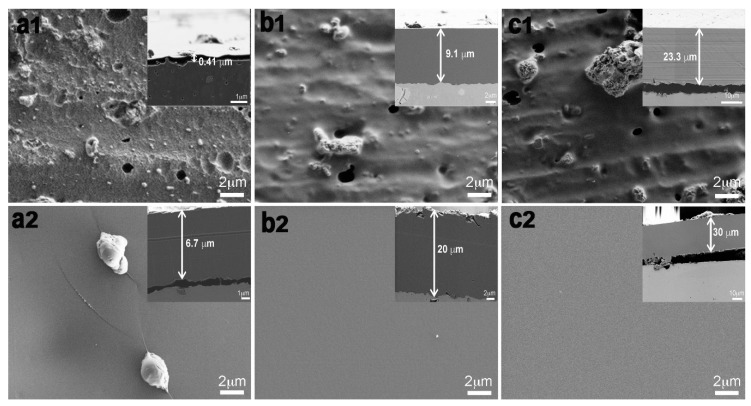
SEM micrographs and cross-sections of the AA2024 alloy coated by radical polymerization treatment, without pseudo-transesterification; (**a1**) AA-PMMA 45′; (**b1**) AA-PMMA 90′; (**c1**) AA-PMMA 120′ and with pseudo-transesterification; (**a2**) AA-PMMA 45′; (**b2**) AA-TE-PMMA 90′; and (**c2**) AA-TE-PMMA 120′.

The poly(methyl methacrylate) [PMMA] coating on the aluminum alloy surface was characterized by scanning electron microscopy (SEM). Figure 3 shows SEM micrographs of the coated alloy surfaces with 45-min, 90-min, and 120-min polymerization treatments. Figure 3(a1,b1,c1) shows the chemically-etched coated alloy without functionalizing with pseudotransesterification. The coating exhibits surface porosity with the presence of cluster-like material. Figure 3(a2,b2,c2) shows the PMMA coatings on the aluminum alloy with the pseudotransesterification treatment. The images show that the surface is smoother and without pores. The insets on each subfigure are images of the cross-section of the coatings.

**Figure 4:**
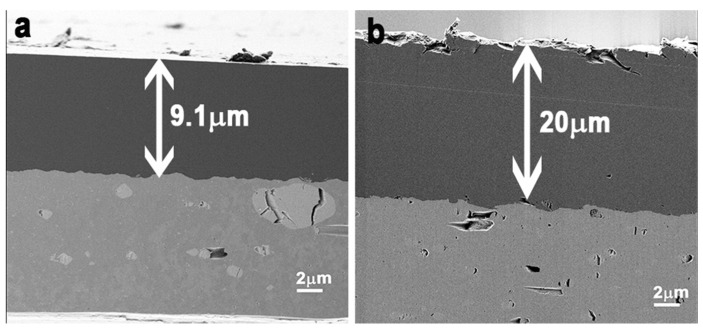
SEM micrographs of the cross sections of the alloy surface and its coatings: (**a**) AA- poly(methyl methacrylate) (PMMA) 90′ and (**b**) AA-TE-PMMA 90′.

Pseudotransesterification promotes polymerization reactions on the alloy with the resulting formation of thicker coatings on the functionalized surface than on the surfaces of untreated alloys, as demonstrated by the results that are presented in Figure 4 for a coated alloy surface with a 90-min polymerization treatment. SEM micrographs of the cross-sections reveal the dependence of the coating formation on the surface pretreatment. It is evident that thicker coatings are formed on the alloy surface that is functionalized by the pseudotransesterification treatment.

### 2.3. Characterization of the Coating Surfaces by XPS

**Figure 5:**
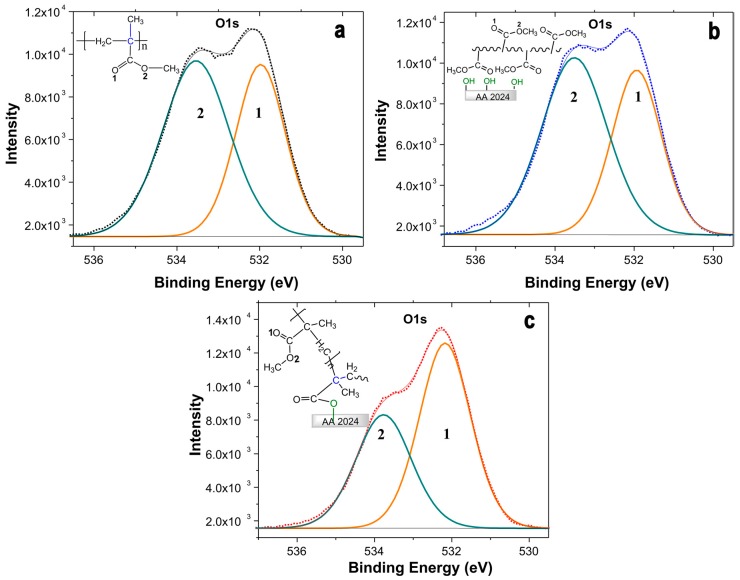
XPS spectra showing the O1s peaks of: (**a**) Poly(methyl methacrylate), (**b**) aluminum alloy coated with poly(methyl methacrylate) (AA-PMMA), and (**c**) aluminum alloy modified with pseudotransesterification and then polymerized with methyl methacrylate (AA-TE-PMMA).

Figure 5 shows the XPS spectra with the O1s peak from the poly(methyl methacrylate) and the O1s peak obtained from the aluminum alloy coated with poly(methyl methacrylate) film.

Table 1 shows the area relationships of the O1s peaks of the different PMMA modifications. Figure 5a shows the non-functionalized PMMA signals where two main components are observed; O=C group (1) at a binding energy of 531.9 eV, and group OC (2) at a binding energy of 533.4 eV. The O=C/OC concentration ratio was extracted from the O1s signals. At 45/56, the ratio was very close to the value of 48/52 in the literature [25,26,27].

The AA-PMMA samples show almost equal amounts of O=C and O-C, as observed in the peaks in Figure 5b, where the O=C/O-C concentration ratio was 42/58, which is very similar to that of the PMMA samples (Figure 5a). In the case of the alloy treated with pseudotransesterification (AA-TE), the O-C bonds gradually decreased, and the O=C bonds increased significantly, as can be seen in Figure 2b, with an O=C/O-C ratio of 57/43. This effect was the same with the aluminum surface modified by pseudotransesterification and subsequently polymerized (AA-TE-PMMA) (Figure 5c), where the highest number of O=C bonds (59%) was detected, and the concentration ratio was 59/41.

The effect of the conformation of polymer chains in the XPS spectra has been investigated experimentally and theoretically [28]. It has been proposed that changes in the orientation of the functional groups of a polymer chain significantly affect the XPS spectra [27]. The pseudotransesterified sample in Figure 5 reveals differences in the signal intensity of 1s oxygen on the surface. The schematic model of the surface molecule (on the right side of the spectrum, Figure 5c) shows a more exposed O=C bond, which could explain why the oxygen intensity of this bond is higher than that of the O-C bond. It is possible that pseudotransesterification influences the growth/order process of the polymer at the surface. In contrast, the growth of the polymer on the non-transesterified sample could be disorderly at the surface, with an exposure in the same proportion of the O=C and O-C oxygen bonds (schematic model, on the left side of the spectrum in Figure 2b).

### 2.4. Contact Angle Measurements

**Figure 6:**
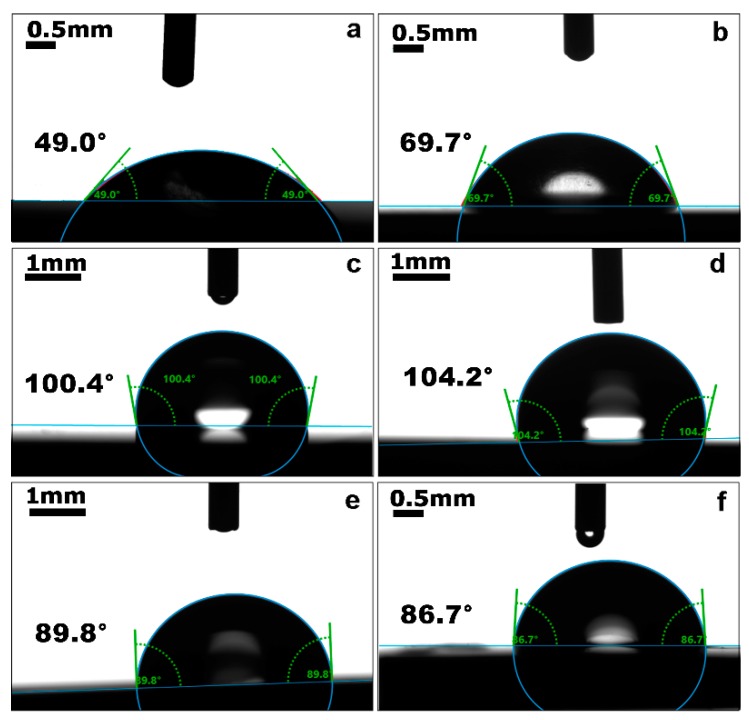
Contact angle of (**a**) AA2024, (**b**) AA2024 + TE, (**c**) AA2024 + PMMA, (**d**) AA2024 + TE + PMMA, (**e**) AA2024 + TE + PMMA-Ag 0.01 wt%, and (**f**) AA2024 + TE + PMMA-Ag 1 wt%.

Figure 6 shows the contact angle profiles obtained for AA2024, AA2024 + TE, AA2024 + TE + PMMA, and PMMA with 0.01 wt% and 1 wt% of silver nanoparticles. It can be observed that AA2024 is the most hydrophilic surface, with the lowest contact angle (49.0°), which increases slightly with the pseudotransesterification treatment (69.7°), while AA2024 coated with PMMA has the highest contact angle values (100.4° and 104.2°), as has been observed in other works [29]. However, no significant influence could be observed of the pseudotransesterification treatment on the hydrophobicity of the coating. The hydrophobic character decreases in the presence of AgNPs, with contact angle values of 89.8° and 86.7° for PMMA with 0.01 wt% and 1 wt% of AgNPs, respectively.

### 2.5. Antibacterial Properties of Coatings

**Figure 7:**
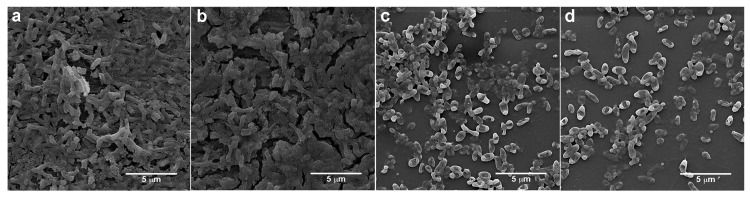
*Pseudomonas aeruginosa* incubated for 24 h on (**a**) AA2024, (**b**) AA2024 + TE, (**c**) AA2024 + PMMA, and (**d**) AA2024 + TE + PMMA.

Figure 7 shows SEM images of Pseudomonas aeruginosa incubated for 24 h on an aluminum alloy without coating (AA2024), an aluminum alloy with treatment (AA2024 + TE), an aluminum alloy with coating (AA2024 + PMMA), and an aluminum alloy with treatment and coating (AA2024 + TE + PMMA). *Pseudomonas aeruginosa* biofilm can be observed on AA2024 and AA2024 + TE. Other authors have reported the formation of biofilm on aluminum, where SEM images have shown bacteria embedded in a layer of extracellular polymeric substances (EPS) on aluminum, which is similar to what can be observed in Figure 7 [30]. In contrast, when *Pseudomonas aeruginosa* is incubated on AA2024 coated with PMMA, the bacterial morphology changes, and EPS is not observed. Studies have shown that Pseudomonas aeruginosa adheres less to hydrophobic than hydrophilic surfaces [31]. The same effects of hydrophilicity/hydrophobicity on bacterial adhesion can be observed in our results.

**Figure 8:**
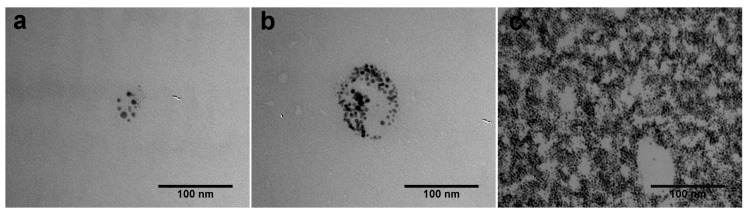
TEM images of (**a**) PMMA-AgNPs 0.01 wt%, (**b**) PMMA-AgNPs 0.1 wt%, and (**c**) PMMA-AgNPs 1 wt%.

To make the PMMA responsive against bacterial proliferation, we incorporated AgNPs into the PMMA coating by in situ polymerization. Figure 8 shows TEM images of the AgNPs in PMMA. AgNPs with sizes between 5–10 nm can be observed, and with good dispersion.

**Figure 9:**
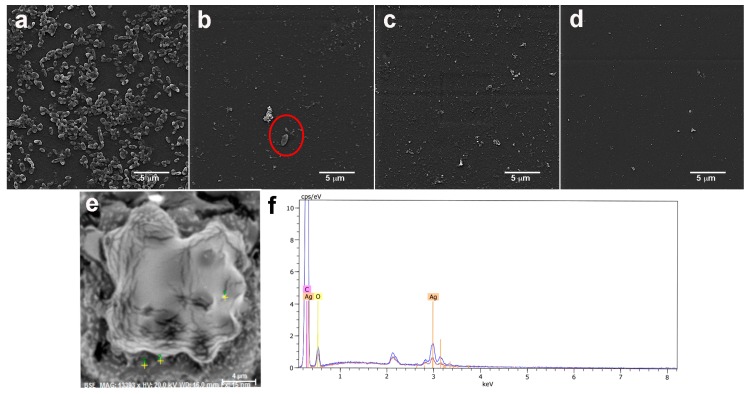
*Pseudomonas aeruginosa* incubated for 24 h on (**a**) AA2024 + TE + PMMA, (**b**) AA2024 + TE + PMMA-Ag 0.01 wt%, (**c**) AA2024 + TE + PMMA-Ag 0.1 wt%, (**d**) AA2024 + TE + PMMA-Ag 1 wt%, (**e**) AA2024 + TE + PMMA-Ag 1 wt% analyzed by EDS (1, 2, 3 correspond to each measurement point), (**f**) EDS spectrum.

The incorporation of AgNPs in the PMMA coating changed the behavior against *Pseudomonas aeruginosa*. We evaluated the antibiofilm capability of the PMMA coating with three quantities of AgNPs; 0.01 wt%, 0.1 wt%, and 1 wt% (Figure 9). The presence of silver in the coating was confirmed by EDS analysis (Figure 9e,f). Few bacteria were observed on PMMA-AgNPs 0.01 wt% (red circle), while no bacteria were observed on PMMA-Ag 0.1 wt% and PMMA-Ag 1 wt%. Additionally, we evaluated the bacterial viability of *Pseudomonas aeruginosa* on aluminum alloy with and without coating. The results showed an inhibition of 99.99% of bacterial colonies for AA2024 + TE + PMMA-Ag containing 0.01 wt% of AgNPs, while that AA2024 + TE + PMMA and AA2024 showed an inhibition of 11.4 wt% and 0.01 wt%, respectively (see Figure S3 in the Supplementary Data).

The antibacterial property of AgNPs is associated with the release of silver ions. Other works have reported that silver ions bind to proteins in the plasma membrane of the bacteria, and alter their permeability. Silver ions also bind to and condense DNA, preventing it from replicating. Another mechanism of silver ions is generating reactive oxygen species (ROS) that alter the metabolic processes of bacteria and the integrity of their membranes [17,32].

### 2.6. Effect of Pseudotransesterification on the Protective Capability of Coatings

**Figure 10:**
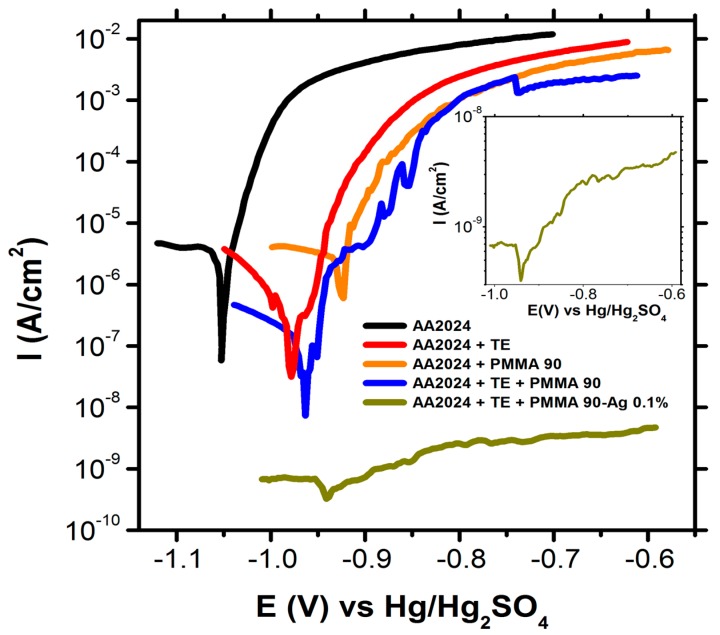
Anodic polarization curves of aluminum alloy surfaces differently treated and coated by polymers in 0.1 M of NaCl at a sweep rate of 0.2 mV s^−1^.

In order to evaluate the protective capacity of the polymeric coatings, the potential current responses of the differently coated aluminum samples were studied in 0.1 M of NaCl and after exposure to electrolytes for 20 h. Figure 10 shows the results.

The corrosion parameters presented in Table 2 were obtained from the Evans diagrams based on the polarization curves in Figure 10.

The anodic curves in Figure 10 show that the current density decreases with pseudotransesterification, which could be related to the increased compaction of the coating (Figure 4). The decreased current density is an order of magnitude lower than the anodic currents of the bare surface, and two orders of magnitude lower than the surfaces coated with the polymer without anchoring. The current density of the coating with AgNPs was three orders of magnitude lower than the bare alloy, reaching a high inhibition efficiency of 0.99%. The large passivation zone that was observed for coatings with AgNPs may be associated with the negative charge of the nanoparticles (zeta potential = −38 mV), which generates electrostatic repulsion with the chloride ions in the corrosive medium. The low current density and high passivation region demonstrate that the film provides an efficient barrier against corrosion agents. The relationship between the compaction of the polymer coatings and the quality of the anticorrosive protection is well known [33,34]. Corrosion agents can diffuse through pores or cracks and reach the interface. In these regions, chemical changes can destabilize the polymer locally, and delaminate it. The build-up of stress in these regions can also result in mechanical damage to the polymer. In any case, chemical and mechanical damage results in sites in which aggressive species such as halides or organic acids in aqueous media catalyze the generation of pitting.

## 3. Materials and Methods

### 3.1. Materials

*p*-Toluenesulfonic acid (PTSA, 98%), hydroquinone (HQ, 99%) and calcium hydride (CaH_2_, 95%) were obtained from Aldrich (Santiago, Chile). Methyl methacrylate (MMA), benzoyl peroxide, nitric acid (20% *v*/*v*), and methanol (P.A) were obtained from Merck (Santiago, Chile). The α-alumina was obtained from Reich (Santiago, Chile), and aluminum alloy (AA2024) was provided by the National Aeronautics Company of Chile (Santiago) (ENAER).

### 3.2. Surface Treatment of the AA2024 Aluminum Alloy

The AA2024 aluminum alloy samples were mechanically polished with SiC grinding paper of 600 grits, 800 grits, 1200 grits, and 2400 grits, and then etched chemically according to the following sequence of steps: (i) immersed in a 0.1-M NaOH solution for two minutes; (ii) rinsed in deionized water; and then immediately (iii) immersed in a HNO_3_ 20% *v*/*v* solution for two minutes; (iv) rinsed in deionized water and dried in a cool air stream. All of the solutions were prepared with deionized water.

### 3.3. Pseudotransesterification Process

The AA2024 alloy and α-alumina surfaces were modified in a distillation system. The MMA monomer, the catalyst PTSA, and hydroquinone were added to the distillation system in the presence of the aluminum alloy, which was previously hydroxylated, in the same manner as in the surface treatment of the above-described AA2024 aluminum alloy. The system was heated to 90 °C with stirring for 20 h. The excess MMA was distilled off under reduced pressure. Specimens were then washed with methanol.

### 3.4. Polymerization of Methyl Methacrylate on Aluminum Alloy

The aluminum alloy was coated by mass radical polymerization using the MMA monomer to form a poly(methyl methacrylate) film on the alloy surface (Scheme 1). Prior to the polymerization treatment, the MMA monomer was washed with 5 wt% NaOH solution, and dried with calcium hydride. MMA polymerization was carried out in a Schlenk reactor, loading the previously modified aluminum alloy sample (Section 2.3), prewashed MMA (15 mL), and benzoyl peroxide (0.05 *wt*/*v*%). The system was kept under stirring at 55 °C. In parallel, MMA was polymerized in the absence of the aluminum alloy sample in the same conditions as indicated above. For both cases, average molecular weights were determined by HPLC chromatography using a Hewlett Packard Series 1100 equipment (Waldbronn, Germany) with a refraction index as the detector, THF as the solvent, and a TSK-GEL (7.5 mm × 30 cm) column. The values of Mw and Mw/Mn that were obtained are 445.037 g/mol and 1.2 g/mol, respectively.

### 3.5. AgNPs Synthesis and PMMA-Ag Preparation

AgNPs were prepared according to the method used by Wang [35], where 1 × 10^−3^ M AgNO_3_ solution containing 1 × 10^−4^ M of oleic acid was mixed dropwise with 4 × 10^−4^ M NaBH_4_ solutions. Then, 2 mL of K_2_HPO_4_ was added to 100 mL of AgNPs suspension together with 100 mL of toluene. AgNPs in the organic phase was rotaevaporated and added to a Schlenk reactor (Soviquim, Santiago, Chile) together with a prewashed MMA monomer (5 mL) and benzoyl peroxide (0.05 *wt*/*v*%) for polymerization reaction. The system was kept under stirring at 55 °C. Silver nanoparticles were analyzed in a Philips Tecnai 12 TEM (Philips, Amsterdam, Netherlands).

### 3.6. Surface Analysis

XPS. The chemical states of the aluminum alloy following the treatment were investigated by X-ray photoelectron spectroscopy (XPS), using a Kratos Axis Ultra DLD (Kratos Analytical, Manchester, UK) at a takeoff angle of 45°, with monochromatic Al K_α_ radiation (hν = 1486.6 eV) at 150 W and 1.0 × 10^−8^ mbar at a pass energy of 23.5 eV. The energy resolution that was used was 0.2 eV. Samples were survey scanned for relevant elements (pass energy = 100 eV, energy step = 0.5 eV). The 1s elemental peak was from carbon (285 eV). The data was processed with CasaXPS, version 2.3.17 (House Software, Sanderland, UK).

SEM. The morphologies of the uncoated and coated aluminum alloy surfaces were investigated by scanning electron microscopy (SEM) using a Philips XL30 (Philips, Amsterdam, Netherlands) at an acceleration voltage of 5 kV. Cross-sections of the coated samples were cut by ultramicrotomy to characterize the surface-coating interface.

### 3.7. Antibiofilm Assay

The samples were inoculated with 100 μL of bacterial suspension and incubated at 37 °C for 16 h. The samples were rinsed twice with 0.01 M of sodium cacodylate/0.15 M NaCl buffer at pH 7.0 and fixed with 1% glutaraldehyde for 2 h at room temperature. Finally, the samples were dehydrated in ascending grades of ethanol (30%, 50%, 70%, 80%, 90%, and 100%), after which they were coated with a thin film of Pt/Pd prior to being observed by SEM [19].

### 3.8. Potentiodynamic Measurements

Measurements were taken with a potentiostat-galvanostat (AUTOLAB PGSTAT30, Metrohm, Palm River-Clair-Mel, Fl, USA), and a standard three-compartment cell. Aluminum alloy AA2024 electrode plates were carefully prepared, leaving a 1-cm^2^ area exposed to the electrolyte. The counter electrode was made of high purity platinum (99.99%), and the reference electrode was made of mercury/mercuric sulfate (Hg/Hg_2_SO_4_) (SSE). The polarization curves were obtained in 0.1 M of NaCl, after 20 h of open circuit potential (OCP) stabilization time, at a scanning rate of 0.1 mV/s in the range of −70 to +350 mV versus the open-circuit potential (OCP). The inhibition efficiency (*IE*) of the coatings and functionalized surface was calculated using the following Equation (1):

(1) $$IE\%=\frac{i_{0}-i_{corr}}{i_{0}}\times 100$$

where $i_{0}$ and $i_{corr}$ are the corrosion current densities of the surface and the surface-functionalized coatings, respectively, as determined by analysis of the Tafel plots.

## 4. Conclusions

Pseudotransesterification is a feasible metallic surface pretreatment to form covalent bonds with polymer coatings. XPS spectra reveal that the polymer on the transesterified specimen grows differently from that grown on the surface without pseudotransesterification. Thus, pseudotransesterification promotes polymerization reactions on alloys, resulting in coarser surface coatings. While the PMMA coating allowed the adhesion of Pseudomonas aeruginosa, the presence of AgNPs prevented biofilms from generating and bacteria from adhering. As well, the decrease in the anodic currents of the coatings, as compared to the bare AA2024, demonstrated the corrosion protection capacity of the coatings. Finally, the protective and antibiofilm features of this coating demonstrate that it is suitable for use in a variety of applications.

## Figures and Tables

**Table 1:** O1s peak area of oxygen plasma-treated PMMA.

Sample	O1s (1) O=C (%)	O1s (2) O-C (%)
PMMA	45	56
AA-PMMA	42	58
AA-TE	57	43
AA-TE-PMMA	59	41

**Table 2:** Open circuit potential, current, and inhibition efficiency of metal surfaces under different treatments obtained from Figure 8.

Sample	OCP (V)	I (A/cm^2^)	IE (%)
AA	−1.06 ± 0.01	4.4 × 10^−7^	n.d
AA + TE	−0.97 ± 0.01	4.3 × 10^−8^	90.0
AA + PMMA	−0.93 ± 0.01	5.9 × 10^−7^	n.d
AA + TE + PMMA	−0.95 ± 0.01	7.1 × 10^−9^	98.0
AA2024 + TE + PMMA-Ag 0.1 wt%	−0.94 ± 0.02	4.1 × 10^−10^	99.0

n.d: not determined.

## Supplementary Materials

The following are available online, Figure S1: Schematic diagram showing pseudo-transesterification of the α-alumina particulates with methyl methacrylate (MMA) (* PTSA: *p*-toluenesulfonic acid), Figure S2: Differential pulse voltammetry of different alumina colloid suspensions in 0.1 M NaOH. Colloid M1: 150 ppm of Al_2_O_3_ particles; Colloid M2: 150 ppm of Al_2_O_3_ particles after being immersed in a solution of MMA monomer and washed with methanol; Colloid M3: 150, 450 and 950 ppm of Al_2_O_3_ particles with pseudo-transesterification treatment, Figure S3: Viability of *Pesudomonas aeruginosa* incubated on (**a**) AA2024, (**b**) AA2024 + PMMA and (**c**) AA2024 + TE + PMMA-AgNPs 0.01 wt%.
