# Supplementary material for: Surface Functionalization of an Aluminum Alloy to Generate an Antibiofilm Coating Based on Poly(Methyl Methacrylate) and Silver Nanoparticles
Source: Molecules. 2018 Oct 24;23(11):2747. doi: 10.3390/molecules23112747 (PMC6278379; doi:10.3390/molecules23112747)
Supplement: Supplementary file 1 [file molecules-23-02747-s001.pdf]

Supplementary File

Surface functionalization of an aluminum alloy to generate an antibiofilm coating based on poly(methyl methacrylate) and silver nanoparticles.

Lisa Muñoz,[1,*] Laura Tamayo,[2] Miguel Gulppi,[1] Franco Rabagliati,[1] Marcos Flores,[3] Marcela Urzúa,[2] Manuel Azocar,[1] Jose Zagal,[1] María V. Encinas,[1] Xiaorong Zhou,[4] George Thompson,[4] Maritza Páez.*[1]

[1] Departamento de Química de los Materiales, Facultad de Química y Biología, Universidad de Santiago de Chile, Av. L. B. O'Higgins 3363, Casilla 40, Correo 33, Santiago, Chile.; lisa.munozm@usach.cl, miguel.gulppi.c@usach.cl, franco.rabagliati@usach.cl, manuel.azocar@usach.cl, jose.zagal@usach.cl, maria.encinas@usach.cl

[2] Departamento de Química, Facultad de Ciencias, Universidad de Chile, Las Palmeras 3425, Ñuñoa, Santiago, Chile.; lauratamayo26@gmail.com, maurzua@uchile.cl

[3] Laboratory of Surface and Nanomaterials, Physics Department, Faculty of Physics and Mathematics Science, Universidad de Chile, Beauchef 850, Santiago, Chile.; mflorescarra@ing.uchile.cl

[4] Corrosion and Protection Centre, School of Materials, The University of Manchester, Manchester M13 9PL, UK.; george.thompson@manchester.ac.uk, xiaorong.zhou@manchester.ac.uk

* Correspondence: lisa.munoz@usach.cl; Tel.: +56-22-718-3426

Differential pulse voltammetry (DPV). In order to verify the pseudotransesterification of the aluminum alloy surface, α-alumina (0,3μm) powder was used as the benchmark for the surface oxide layer on the aluminum alloy. Three different samples of α-alumina powder were prepared: for the first (M1) sample, the powder was washed with methanol and dried in vacuum. For the second (M2) sample, the powder was wetted with MMA and subsequently washed with methanol and dried under vacuum. For the third sample (M3), the powder was prepared following the protocol described in Section 2.3. From the three different alumina powder samples (M1, M2, and M3), colloidal suspensions were prepared in 0.1 M of NaOH. Differential pulse voltammetry (DPV) of three alumina colloidal suspensions were performed using a potentiostat CHI 1140B with the following electrodes: a disc of pyrolytic graphite ordinary 0.192 cm^2 as the working electrode; a platinum wire of 8.0 cm^2 as the counter electrode; and a saturated calomel electrode as the reference electrode. The (α-alumina/ 0.1M NaOH) colloidal system was bubbled with nitrogen gas at room temperature.

Pseudotransesterification on α-alumina particulate.

In order to demonstrate that the alumina surface is functionalized via pseudotransesterification with MMA (Figure 3), we treated finely divided α-alumina particulate (0.3-μm diameter) following the procedure described in Section 2.3.

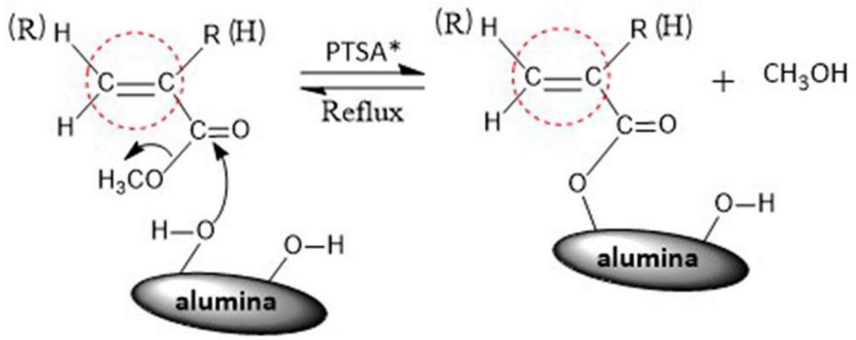

Figure S1. Schematic diagram showing pseudotransesterification of the α-alumina particulates with methyl methacrylate (MMA) (* PTSA: p-toluenesulfonic acid).

Changes resulting from the pseudotransesterification process can be seen in the differential pulse voltammetry (DPV) of the three alumina colloidal suspensions that are shown in Figure 4. Comparison of the E/I responses of the colloidal system reveals differences, in particular a current peak at -0.54 V (SCE) (sample M3), which is associated with the oxidation of the double bond that is present in the MMA monomer molecule.[21] The current peak on the curve for M3 increases linearly with the amount of the treated alumina in the suspension, indicating oxidation of species on the alumina with pseudotransesterification treatments. During the cathodic sweep, two current peaks at –0.51 V and –0.83 V are evident on the curve for M3, which increases in intensity with the increasing amount of the treated alumina. These peaks may be associated with a reduction of carbonyl carbon,[22,23] which is not observed for the colloidal system containing alumina without the pseudotransesterification treatment. Further, current peaks are not associated with adsorbed MMA since DPV measurements of M2 did not reveal the current peaks described above, indicating that the weakly adsorbed monomer was removed during washing. Thus, the differential pulse voltammetry shows clearly that the pseudotransesterification has occurred on α-alumina particulates during the pseudotransesterification treatment.

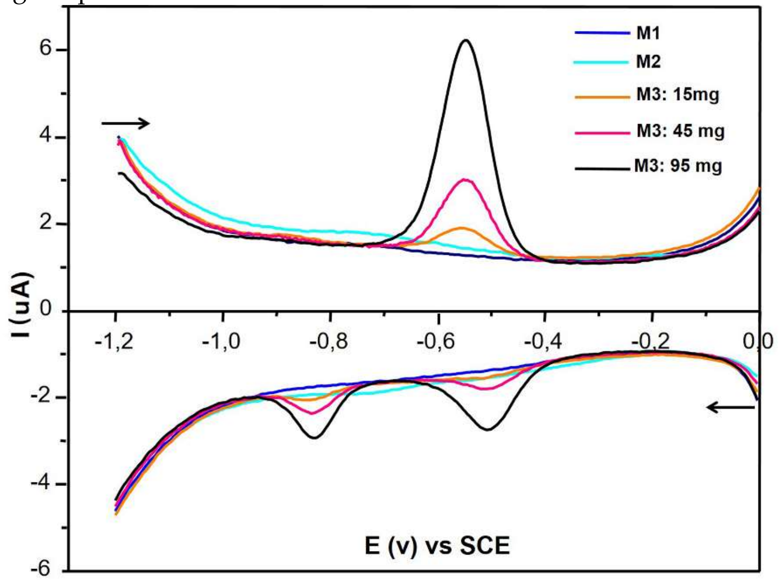

Figure S2. Differential pulse voltammetry of different alumina colloid suspensions in 0.1 M of NaOH. Colloid M1: 150 ppm of Al_2O_3 particles; Colloid M2: 150 ppm of Al_2O_3 particles after being

immersed in a solution of MMA monomer and washed with methanol; Colloid M3: 150 ppm, 450 ppm, and 950 ppm of Al_2O_3 particles with pseudotransesterification treatment.

Bacterial Viability

The antibacterial ability was assessed against *Pesudomonas aeruginosa*. One drop of 10^6 CFU/ml bacterial suspension was deposited on the surface of the AA2024, AA2024 + PMMA, and AA2024 + PMMA-AgNPs0.01%, of dimensions 3 × 1 cm^2, and incubated for 16 h. Samples were washed with 10 ml of a solution containing 0.88 wt.% NaCl and 1 wt.% Tween 80. This was undertaken in order to drag the bacteria incubated in the surface of the nanocomposite. Subsequently, 40 µl of the solution with entrained bacteria was transferred to a nutrient medium, Mueller Hinton agar, and incubated for 16 h at 37 °C. After incubation, colonies were counted. The experiments were repeated four times. Bacterial inhibition was calculated by the following equation:

Bacterial inhibition (%) = C_i*100/C_0

Where C_0 corresponds to the bacterial colonies obtained of AA2024, and Ci corresponds to the bacterial colonies obtained of AA2024 with a different coating.

Figure S3 showed an inhibition of 99.99% of bacterial colonies for AA2024 + TE + PMMA-AgNPs0.01%, while that of AA2024 + TE + PMMA y AA2024 showed inhibitions of 11.4% and 0.01%, respectively. Clearly, the surface of the aluminum alloy does not show an antibacterial effect against *Pseudomonas aeruginosa*, obtaining a low inhibition of bacterial colonies (0.01%), while the PMMA coating shows a slight reduction of the colonies (11.4%). As observed in the SEM images (Figure 9 in the manuscript), PMMA has an inhibitory effect on the production of biofilm, which could affect the viability of *Pseudomonas aeruginosa*. On the other hand, a PMMA coating containing 0.01 wt.% of AgNPs shows 99.99% of the inhibition of bacterial colonies, demonstrating the excellent antibacterial properties of AgNPs, even when a low amount was used.

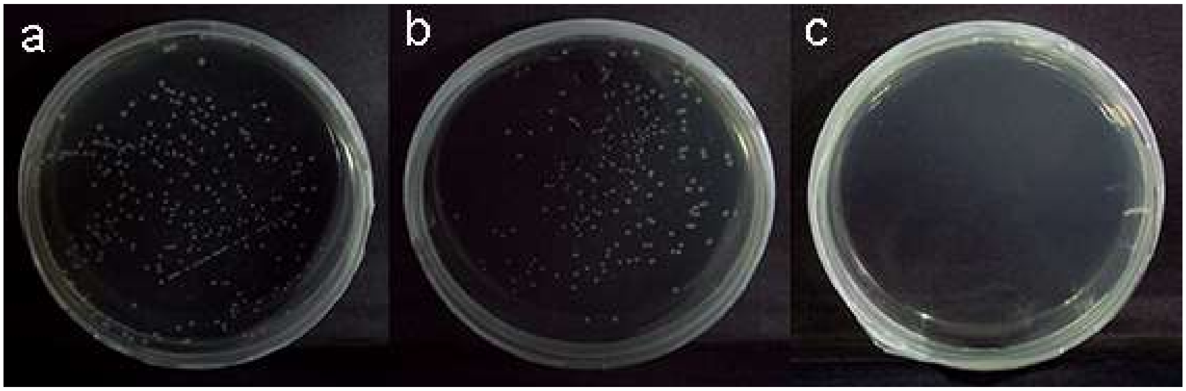

Figure S3. Viability of *Pesudomonas aeruginosa* incubated on (a) AA2024, (b) AA2024 + PMMA, and (c) AA2024 + TE + PMMA-AgNPs 0.01%.
